# Enhancing entrepreneurial intention through critical thinking disposition and positive psychological traits: evidence from Chinese higher education

**DOI:** 10.3389/fpsyg.2026.1823907

**Published:** 2026-04-13

**Authors:** Zhicong Zhang, Zonglong Li, Xiuge Liu, Hua Shao

**Affiliations:** 1 School of Educational Science, Quanzhou Normal University, Quanzhou, China; 2 School of Foreign Languages, Quanzhou Normal University, Quanzhou, China

**Keywords:** critical thinking disposition, entrepreneurial intention, entrepreneurial self-efficacy, entrepreneurship education, higher education, positive psychological traits

## Abstract

University students’ entrepreneurial intention (EI) is a key focus of entrepreneurship education and policy, especially in China’s context of promoting innovation-driven development. This study explores how critical thinking disposition (CTD), positive psychological traits (PPT), and entrepreneurial self-efficacy (ESE) jointly influence EI. Using structural equation modeling and latent variable interaction analysis, Study 1 surveyed vocational college students and found that CTD significantly predicted EI, without PPT moderating this effect. Study 2, conducted with undergraduate students, introduced ESE as a mediator. Data analysis using bootstrapping procedures revealed that CTD predicted ESE, which in turn predicted EI. The interaction between CTD and PPT also significantly predicted ESE, supporting a moderated mediation model. This research contributes to the entrepreneurship literature by uncovering the synergistic cognitive-psychological mechanisms driving EI. The findings provide a novel theoretical framework and practical insights for entrepreneurship education, highlighting the necessity of simultaneously cultivating cognitive skills and psychological resources to enhance students’ entrepreneurial potential.

## Introduction

Universities are increasingly involved in science- and technology-driven economic development, with many institutions adopting an entrepreneurial orientation that supports student ventures and innovation (Feola et al., 2021). In response, entrepreneurship education has expanded globally, with the aim of developing students’ knowledge, skills, and creative capacities (Talukder et al., 2024a). In China, this trend is reflected in national policies that position entrepreneurship as a means of promoting innovation and addressing graduate unemployment (Lv et al., 2021). Entrepreneurship education has been incorporated into vocational and undergraduate programs, often with an emphasis on experiential learning and applied business skills (Zhou et al., 2024). However, the presence of formal training and institutional support does not necessarily lead to corresponding entrepreneurial outcomes. For example, the rate of entrepreneurship among graduates of Chinese vocational colleges remains below 2%, which is considerably lower than that observed among graduates from regular universities (Wu and Tian, 2022). This gap indicates that external support alone is insufficient and highlights the need to examine the internal factors that shape students’ motivation to engage in entrepreneurship.

Recent research suggests that the effects of educational and environmental support on entrepreneurial intention (EI) depend on a set of underlying psychological mechanisms, including attitudes, perceived behavioral control, and individual traits (Talukder et al., 2024b, 2025). Although prior studies have often focused on demographic variables or single psychological factors, less attention has been given to how cognitive and psychological processes jointly influence the development of entrepreneurial motivation (Zhang et al., 2025). In particular, the combined role of thinking dispositions and positive psychological characteristics remains underexplored. Existing work has shown that cognitive factors, such as critical thinking disposition (CTD), support the evaluation of business opportunities, yet it is less clear how these cognitive tendencies interact with individuals’ psychological resources. Given the uncertainty and demands associated with entrepreneurial activity, cognitive capacities alone may not be sufficient. Positive psychological traits (PPT), such as resilience, optimism, and perseverance, may be necessary to sustain effort and manage challenges. It remains unclear whether PPT functions as a moderating factor that shapes the extent to which CTD contributes to entrepreneurial self-efficacy and, in turn, entrepreneurial intention.

This study draws on the Theory of Planned Behavior (Ajzen, 1991), which conceptualizes EI as a planned behavior influenced by attitudes, social norms, and perceived behavioral control, often represented by self-efficacy. Social Cognitive Career Theory similarly identifies self-efficacy as a key mechanism underlying goal-directed behavior (Lent et al., 1994; Munir et al., 2024). In addition, Seligman’s PERMA framework and the EPOCH model provide a basis for conceptualizing PPT (Kern et al., 2016; Seligman, 2018). Based on these perspectives, the present study examines the relationships among CTD, PPT, entrepreneurial self-efficacy (ESE), and EI in a sample of Chinese college students. It focuses on whether CTD predicts EI, whether this relationship operates through ESE, and whether PPT conditions the association between CTD and ESE, thereby clarifying how cognitive and psychological factors jointly contribute to entrepreneurial intention.

## Literature review and hypotheses development

### Theoretical foundations

The conceptual framework of this research is grounded in the Theory of Planned Behavior (TPB) (Ajzen, 1991) and Social Cognitive Career Theory (SCCT) (Lent et al., 1994). According to the TPB, entrepreneurial intention (EI) is shaped by attitudes, subjective norms, and perceived behavioral control, which is commonly operationalized as entrepreneurial self-efficacy (ESE) in the entrepreneurship context. Complementarily, SCCT highlights self-efficacy as a central cognitive mechanism that translates individual capabilities into goal-directed actions. While these theories provide a robust foundation for understanding intention formation, they often overlook the specific higher-order cognitive dispositions and psychological resources required to navigate the high uncertainty of entrepreneurship. By integrating TPB and SCCT, this study constructs a moderated mediation model to systematically examine how cognitive and psychological factors jointly shape students’ EI.

### Hypothesis development

#### Critical thinking disposition (CTD) and entrepreneurial intention (EI)

Recently, increasing attention has been directed toward the association between higher-order thinking and EI. As a key antecedent of entrepreneurial behavior, EI reflects an individual’s planned commitment and psychological readiness to engage in entrepreneurial activities (Krueger et al., 2000). CTD, as a form of higher-order thinking, refers to a relatively stable tendency to engage in logical analysis, evaluative judgment, and reflective reasoning when dealing with problems or information (Facione et al., 1995; Fan and See, 2022). Unlike discrete cognitive skills, CTD captures an individual’s willingness to think carefully and critically in situations characterized by uncertainty, which is a defining feature of the entrepreneurial process.

The relationship between CTD and EI can be understood in terms of the cognitive demands associated with entrepreneurship. Entrepreneurial activities require individuals to identify opportunities, assess risks, and make decisions based on available evidence. A stronger critical thinking disposition facilitates these processes by enabling individuals to process complex information, anticipate potential challenges, and develop appropriate strategies, all of which contribute to entrepreneurial readiness. Recent research has moved beyond simple empirical associations and highlights the relevance of critical thinking for feasibility assessment. For example, studies show that critical thinking improves students’ ability to evaluate business opportunities systematically and interpret entrepreneurial information, which in turn supports their intention to start a business (Zhang et al., 2025). In addition, instructional approaches that emphasize creative problem solving, design thinking, and critical inquiry have been found to predict EI by fostering reflective thinking and the redefinition of problems (Nguyen and Do, 2021; Paiva et al., 2021; Woraphiphat and Roopsuwankun, 2023). Taken together, these findings suggest that individuals with higher levels of CTD are more likely to identify viable opportunities and assess risks effectively, thereby strengthening their entrepreneurial intention. Accordingly, the following hypothesis is proposed:

*H1*: Critical thinking disposition is positively associated with entrepreneurial intention.

#### The mediating role of entrepreneurial self-efficacy (ESE)

Although CTD provides a cognitive basis for entrepreneurship, the SCCT suggests that distal cognitive traits tend to influence behavioral intentions through more proximal self-regulatory mechanisms, particularly ESE (Fuller et al., 2018; Kumar and Shukla, 2019; Wang et al., 2023). ESE refers to an individual’s belief in their ability to perform entrepreneurial tasks and fulfill related roles. From a theoretical perspective, the cognitive processes associated with CTD, including evidence evaluation, questioning of assumptions, and perspective taking, contribute to the development of self-efficacy. Through engagement in reflective thinking and logical analysis, students can better understand the complexity of entrepreneurial processes, break down challenges into manageable components, and develop workable problem-solving strategies. Such experiences are likely to strengthen their confidence in their entrepreneurial capabilities, thereby enhancing ESE.

ESE, in turn, plays an important role in shaping entrepreneurial intention. Within the TPB framework, it is widely regarded as a key predictor of EI, linking cognitive capacities to perceptions of feasibility (Ciuchta and Finch, 2019; Erikson, 2002; Newman et al., 2019). Empirical studies indicate that individual traits, such as cognitive flexibility, need for achievement, and grit, often influence EI indirectly through their effects on self-efficacy beliefs (Al-Qadasi et al., 2023; Mishra and Singh, 2024; Uysal et al., 2022; Zhang and Chen, 2024). More recent scholarship has moved beyond simple mediation to highlight the conditional role of psychological and contextual resources. Factors including psychological capital, social norms, family support, and competitive contexts can moderate the ESE–EI pathway, implying that ESE operates within a broader system of interacting influences (Balgiu et al., 2025; Munir et al., 2024; Saoula et al., 2023; Wang et al., 2023). Across diverse cultural contexts, evidence consistently shows that ESE predicts EI directly while also mediating the impact of entrepreneurship education, personality traits, and psychological resources (Liu et al., 2019; Wang et al., 2023; Wu et al., 2022).

Following this line of reasoning, CTD may increase students’ sense of competence in entrepreneurial contexts, which in turn supports their intention to engage in entrepreneurial activity. Accordingly, the following hypothesis is proposed:

*H2*: Entrepreneurial self-efficacy mediates the relationship between critical thinking disposition and entrepreneurial intention.

#### The moderating role of positive psychological traits

Although CTD contributes to the development of ESE, the extent to which cognitive analysis translates into self-efficacy may depend on individuals’ psychological characteristics. Positive psychological traits (PPT), including optimism, resilience, perseverance, and connectedness (Kern et al., 2016), may therefore play a moderating role in the relationship between CTD and ESE.

This moderating effect can be understood in relation to the demands of entrepreneurial contexts. Entrepreneurship involves uncertainty and the possibility of failure. While critical thinking enables individuals to identify risks and potential obstacles, extensive analytical processing may also increase hesitation or self-doubt, particularly when psychological resources are limited. In this regard, PPT may help individuals manage the emotional demands associated with risk evaluation. From a resource-based perspective, traits such as optimism and resilience can support individuals in interpreting identified risks as manageable and in maintaining engagement with challenging tasks (Balgiu et al., 2025; Chen, 2024).

The role of PPT may therefore differ across individuals. For those with higher levels of PPT, the insights derived from critical thinking are more likely to contribute to stronger self-efficacy, as these individuals are better able to maintain confidence and persistence in the face of anticipated difficulties. In contrast, for individuals with lower levels of PPT, detailed risk appraisal associated with CTD may increase concerns about failure and reduce confidence in their ability to act, thereby limiting the development of ESE (Haddoud et al., 2024; Jin, 2017).

In this sense, PPT may shape the extent to which critical thinking supports the formation of entrepreneurial self-efficacy. Accordingly, the following hypothesis is proposed:

*H3*: Positive psychological traits moderate the relationship between critical thinking disposition and entrepreneurial self-efficacy, such that this relationship is stronger at higher levels of positive traits.

### Overview of the present research

To address the research objectives, this study adopts a two-part design. Instead of testing a complex model within a single sample, which may limit generalizability and increase the risk of common method bias, a stepwise approach was used across different educational contexts.

Study 1 examines the conditions under which CTD is related to EI, with a focus on the moderating role of PPT. The sample consists of vocational college students, whose training emphasizes practical skills and provides a context in which students are more directly exposed to applied entrepreneurial activities.

Study 2 builds on these findings by examining the underlying mechanism. ESE is introduced to test a moderated mediation model, allowing us to assess both whether and how CTD is associated with EI. This study uses a sample of undergraduate students, whose educational experience is more academically oriented. Including this group allows for an examination of whether the proposed relationships are observed across different educational settings.

After reviewing the relevant literature and theories, we propose a comprehensive conceptual model to test our hypotheses. The integrated model employs ESE as a mediator to connect CTD to EI, and PPT as a moderator in the link between CTD and ESE.

## Study 1

### Methods

#### Participants

The study employed a convenience sampling method to survey students from several higher vocational colleges in a selected region. To enhance sample representativeness and mitigate potential sampling bias, participants were recruited from multiple institutions rather than a single college. The research team collaborated with college administrators and instructors to distribute an online survey link (via platforms such as Wenjuanxing) through official class communication groups. Participation was entirely voluntary, and informed consent was obtained prior to the survey. To reduce social desirability bias, participants were assured that their responses would remain strictly anonymous and be used solely for academic purposes. Furthermore, to ensure data quality, attention-check items were embedded in the questionnaire, and responses with missing data, unusually short completion times, or straight-lining patterns were excluded.

Following this procedure, 1,374 valid questionnaires collected. In terms of registered permanent residence (hukou), 71% of participants held rural hukou (*n* = 975) while 29% held urban hukou (*n* = 399). The sample consisted of 52.8% male students (*n* = 726) and 47.2% female students (*n* = 648). By year of study, freshman accounted for 56.5% (*n* = 776), sophomores 34.4% (*n* = 472), and juniors 9.5% (*n* = 126). Regarding academic disciplines, STEM majors accounted for 39.7% (*n* = 546), liberal arts majors 31.6% (*n* = 434), and arts majors comprised the remaining proportion of the sample.

### Measures

#### Positive psychological traits

Students’ PPT were assessed using the EPOCH Measure of Adolescent Wellbeing developed by Kern et al. (2016), based on Seligman’s PERMA model of wellbeing. The Chinese-translated version validated by Dong et al. (2018) was employed in this study. The scale comprises 20 items. Responses were rated on a 5-point Likert scale ranging from 1 (*not at all like me*) to 5 (*very much like me*), with higher scores indicating greater levels of PPT.

The scale demonstrated excellent internal consistency in the current sample (Cronbach’s *α* = 0.950; 95% CI = [0.946, 0.954]). Confirmatory factor analysis (CFA) indicated an acceptable model fit: *χ*^2^(170) = 2019.690, *χ*^2^/df = 11.880, RMSEA = 0.089 (90% CI [0.086, 0.092]), CFI = 0.836, TLI = 0.817, SRMR = 0.057.

#### Entrepreneurial intention

EI was measured using a 5-item scale developed by Li et al. (2008), grounded in the TPB. Each item was rated on a 5-point Likert scale, with higher scores reflecting stronger EI.

The internal reliability of the scale was high (Cronbach’s *α* = 0.891; 95% CI = [0.882, 0.900]). CFA results supported the construct’s validity: *χ*^2^(5) = 35.543, *χ*^2^/df = 7.108, RMSEA = 0.067 (90% CI [0.047, 0.088]), CFI = 0.985, TLI = 0.970, SRMR = 0.020.

#### Critical thinking disposition

The CTD was assessed using the College Students’ Critical Thinking Disposition Scale developed by Huang (2015). The scale includes 22 items across four dimensions: widespread doubt, self-reflection, open-mindedness, and careful thinking. Responses were given on a 5-point scale ranging from 1 (*strongly disagree*) to 5 (*strongly agree*), with higher scores reflecting stronger CTD.

The scale demonstrated excellent reliability in this study (Cronbach’s *α* = 0.928; 95% CI = [0.923, 0.934]). CFA indicated a good model fit: *χ*^2^(209) = 1299.345, *χ*^2^/df = 6.217, RMSEA = 0.062 (90% CI [0.058, 0.065]), CFI = 0.875, TLI = 0.862, SRMR = 0.063.

#### Data analysis

All statistical analyses for Study 1 were conducted using IBM SPSS Statistics 24.0 and Mplus Version 8.3. Descriptive statistics, reliability analyses (Cronbach’s *α* with 95% confidence intervals), and Pearson correlation coefficients were computed using SPSS to assess the basic properties of the measured variables. CFA were performed in Mplus to conduct the preliminary test for common method bias.

We employed path analysis using observed variable scores (manifest variables) in Mplus to test the moderating effect. Model fit evaluated using standard indices: *χ*^2^/df, RMSEA, CFI, TLI, and SRMR. Acceptable thresholds were defined as RMSEA < 0.08, CFI and TLI > 0.90, and SRMR < 0.08 (Hu and Bentler, 1999). All continuous predictors were mean-centered prior to the construction of interaction terms to reduce multicollinearity. Furthermore, to evaluate the strength of relationships and the explanatory power of the model, standardized path coefficients ( β ) and $R^{2}$ values were calculated and reported. Bootstrapping procedures with 5,000 resamples were used to obtain 95% bias-corrected confidence intervals for the interaction effect. A confidence interval that did not include zero was interpreted as evidence of a statistically significant moderation.

### Results

#### Common method variance test

To robustly assess the potential influence of common method bias (CMB), several statistical procedures were conducted following prior recommendations (Podsakoff et al., 2003). First, Harman’s single-factor test was performed using unrotated principal component analysis (Cooper et al., 2020). The first factor accounted for 29.33% of the total variance, which is below the commonly used threshold of 40%.

Second, a single-factor confirmatory factor analysis (CFA) was conducted. The model showed poor fit to the data (*χ*^2^/df = 22.45, CFI = 0.77, TLI = 0.79, RMSEA = 0.16), suggesting that a single latent factor does not adequately explain the observed variance.

Third, the unmeasured latent method construct (ULMC) approach was applied by adding a common method factor to the measurement model. Model comparisons indicated that including this factor did not meaningfully improve model fit (Δ*χ*^2^/df = 0.03, ΔCFI = 0.01, ΔTLI = 0.01, ΔRMSEA = 0.00). The changes in fit indices remained below the 0.02 criterion, indicating that the addition of the method factor did not substantially alter the model (Podsakoff et al., 2003; Simmering et al., 2014). Taken together, these results suggest that CMB is unlikely to have a substantial impact on the findings.

#### Descriptive statistics and correlation analysis

Pearson correlation analysis was conducted to examine the relationships among the key study variables (*N* = 1,374). The PPT (*M* = 3.53, *SD* = 0.75) were significantly and positively correlated with CTD (*M* = 3.69, *SD* = 0.57), *r* = 0.564, *p* < 0.01. PPT also showed a strong positive correlation with EI (*M* = 3.42, *SD* = 0.73), r = 0.750, *p* < 0.01. In addition, CTD was significantly positively associated with EI, r = 0.562, *p* < 0.01.

#### Test of moderating effect

Path analysis was employed to test the moderating effect, with CTD as the independent variable, EI as the dependent variable, and PPT as the moderator, while controlling for relevant covariates (e.g., gender, year of study). Because this path model was fully saturated (i.e., zero degrees of freedom), it yielded a perfect fit to the data. The model accounted for 38.4% of the variance in EI ( $R^{2}=0.384,\mathrm{SE}=0.025$ ).

Specifically, CTD significantly predicted EI (*β* = 0.438, *SE* = 0.032, *t* = 13.478, *p* < 0.001, 95% CI [0.374, 0.500]). PPT also exerted a significant positive effect on EI (*β* = 0.179, *SE* = 0.034, *t* = 5.250, *p* < 0.001, 95% CI [0.113, 0.249]). However, the interaction between CTD and PPT was not statistically significant (*β* = 0.037, *SE* = 0.027, *t* = 1.340, *p* = 0.180, 95% CI [−0.008, 0.068]), suggesting that the moderating effect was not supported. The overall model accounted for 38.4% of the variance in EI.

To further probe the potential moderating effect, conditional effects were examined at three levels of PPT (−1 SD, mean, and +1 SD). The effect of CTD on EI ranged from 0.414 at low levels of positive traits to 0.462 at high levels. However, the 95% confidence intervals overlapped across all levels, indicating that the slope differences were not statistically significant. Thus, in this study, PPT did not significantly moderate the relationship between CTD and EI.

## Study 2

### Methods

#### Participants

The study employed a convenience sampling method to survey students from several higher colleges in a selected region. Following a rigorous procedure similar to Study 1, participants were recruited through a collaborative effort with university counselors and instructors. The online survey was distributed across various departments to ensure a diverse representation of academic backgrounds. To minimize the inherent limitations of convenience sampling, we intentionally targeted students across all 4 years of study and diverse disciplines, thereby improving the overall representativeness of the sample. All participants were informed of the study’s purpose, the voluntary nature of their participation, and the strict anonymity of their data. Rigorous data screening procedures were also applied to remove invalid responses (e.g., failed attention checks or uniform response patterns).

Consequently, 779 valid questionnaires collected. In terms of hukou, 64.7% of participants held rural hukou (*n* = 504) while 35.3% held urban hukou (*n* = 275). The sample consisted of 36.7% male students (*n* = 286) and 63.3% female students (*n* = 493). By year of study, freshman accounted for 29.3% (*n* = 228), sophomores 31.3% (*n* = 244), juniors 22.7% (*n* = 177), and seniors 16.7% (*n* = 130). Regarding academic disciplines, STEM majors accounted for 60.5% (*n* = 471), liberal arts majors 29.8% (*n* = 232), and arts majors comprised the remaining proportion of the sample.

### Measures

#### Entrepreneurial self-efficacy

ESE was measured using a 14-item scale developed by Shi (2018), based on the work of Lucas and Cooper (2005) and Tang (2009). The scale consists of three subdimensions: Innovation Efficacy, Opportunity Recognition Efficacy, and Relationship Coordination Efficacy. All items were rated on a 5-point Likert scale ranging from 1 (*strongly disagree*) to 5 (*strongly agree*), with higher scores reflecting greater confidence in performing entrepreneurial tasks.

In this study, the scale demonstrated excellent internal consistency (Cronbach’s *α* = 0.948; 95% CI = [0.943, 0.953]). Confirmatory factor analysis indicated good model fit: *χ*^2^(77) = 367.704, *χ*^2^/df = 4.775, RMSEA = 0.070 (90% CI [0.063, 0.077]), CFI = 0.932, TLI = 0.920, SRMR = 0.040.

#### Entrepreneurial intention

EI was measured using the same scale applied in Study 1 (Li et al., 2008). In the present study, the scale again demonstrated strong reliability (Cronbach’s *α* = 0.912; 95% CI = [0.901, 0.921]). CFA supported the model’s structural validity: *χ*^2^(5) = 32.070, *χ*^2^/df = 6.414, RMSEA = 0.085 (90% CI [0.057, 0.112]), CFI = 0.981, TLI = 0.963, SRMR = 0.020.

#### Positive psychological traits

PPT were assessed using the EPOCH Measure of Adolescent Wellbeing, as in Study 1 (Kern et al., 2016; Dong et al., 2018). In the current sample, the internal reliability was excellent (Cronbach’s *α* = 0.953; 95% CI = [0.948, 0.957]). CFA results indicated acceptable model fit: *χ*^2^(170) = 1409.994, *χ*^2^/df = 8.294, RMSEA = 0.097 (90% CI [0.092, 0.102]), CFI = 0.835, TLI = 0.816, SRMR = 0.058.

#### Critical thinking disposition

The same measure of CTD used in Study 1 (Huang, 2015). Internal consistency remained high (Cronbach’s *α* = 0.933; 95% CI = [0.926, 0.940]). Confirmatory factor analysis indicated a satisfactory model fit: *χ*^2^(209) = 906.463, *χ*^2^/df = 4.337, RMSEA = 0.066 (90% CI [0.061, 0.070]), CFI = 0.884, TLI = 0.871, SRMR = 0.056.

#### Data analysis

The basic data analysis procedures in Study 2 were consistent with Study 1. Further analyses were conducted using SPSS 24.0 and Mplus 8.3, focusing on testing the mediating role of ESE and the moderated mediation effect of PPT. Specifically, we employed path analysis with observed variable scores in Mplus to test the proposed hypotheses. All continuous variables were mean-centered to reduce multicollinearity. First, we tested a direct effect model to examine whether CTD predicted EI. Next, a moderated model was constructed to test the interaction effect.

Finally, a moderated mediation model was estimated to examine whether ESE mediated the relationship between CTD and EI, and whether this indirect pathway was moderated by PPT. The MODEL INDIRECT and MODEL CONSTRAINT commands in Mplus were used to compute indirect effects and test for moderated mediation. Consistent with Study 1, standardized path coefficients (*β*) and R^2^ values were reported to demonstrate the explanatory power of the structural models. A bias-corrected bootstrap method with 5,000 resamples was employed to estimate the confidence intervals of both indirect and conditional effects.

### Results

#### Common method variance test

Following the same rigorous procedures as in Study 1, we assessed CMB using multiple approaches. Harman’s single-factor test showed that the first factor explained 37.52% of the variance, which is below the 40% threshold. Furthermore, a single-factor CFA demonstrated a poor fit to the data (*χ*^2^ /df = 23.73, CFI = 0.72, TLI = 0.69, RMSEA = 0.19). We then introduced an unmeasured common method latent factor to the structural model. The changes in fit indices between the model with and without the method factor were negligible (Δ*χ*^2^/df = 0.04, ΔCFI = 0.01, ΔTLI = 0.01, ΔRMSEA = 0.01). Because the improvements in model fit were all below 0.02, we conclude that CMB is not a significant concern in this study (Podsakoff et al., 2003; Simmering et al., 2014).

#### Descriptive statistics and correlation analysis

Pearson correlation analysis was conducted to examine the relationships among the key study variables (*N* = 779). ESE (*M* = 3.37, *SD* = 0.74) was significantly and positively correlated with EI (*r* = 0.630, *p* < 0.01), CTD (*r* = 0.702, *p* < 0.01), and PPT (*r* = 0.666, *p* < 0.01). EI (*M* = 3.04, *SD* = 0.89) was also positively correlated with CTD (*r* = 0.500, *p* < 0.01) and PPT (*r* = 0.478, p < 0.01). Moreover, CTD (*M* = 3.65, *SD* = 0.61) and PPT (*M* = 3.50, *SD* = 0.76) showed a strong positive correlation (*r* = 0.709, *p* < 0.01).

#### Test of mediating and moderating effect

To examine the mediating role of ESE, regression analyses were conducted with CTD as the independent variable and EI as the dependent variable. Similar to Study 1, this mediation model was fully saturated, yielding a perfect fit. The model explained 40.4% of the variance in EI ( $R^{2}=0.404,\mathrm{SE}=0.029$ ). Results indicated that CTD significantly predicted ESE (*β* = 0.702, *SE* = 0.024, *t* = 29.301, *p* < 0.001, 95% CI [0.652, 0.764]), and both CTD (*β* = 0.143, *SE* = 0.041, *t* = 3.503, *p* < 0.001, 95% CI [0.063, 0.223]) and ESE (*β* = 0.515, *SE* = 0.038, *t* = 13.580, *p* < 0.001, 95% CI [0.442, 0.590]) significantly predicted EI. The indirect effect of CTD on EI through ESE was significant (*β* = 0.358, *SE* = 0.032, *t* = 11.289, *p* < 0.001, 95% CI [0.297, 0.421]), accounting for 71.45% of the total effect, suggesting partial mediation.

**Figure 1:**
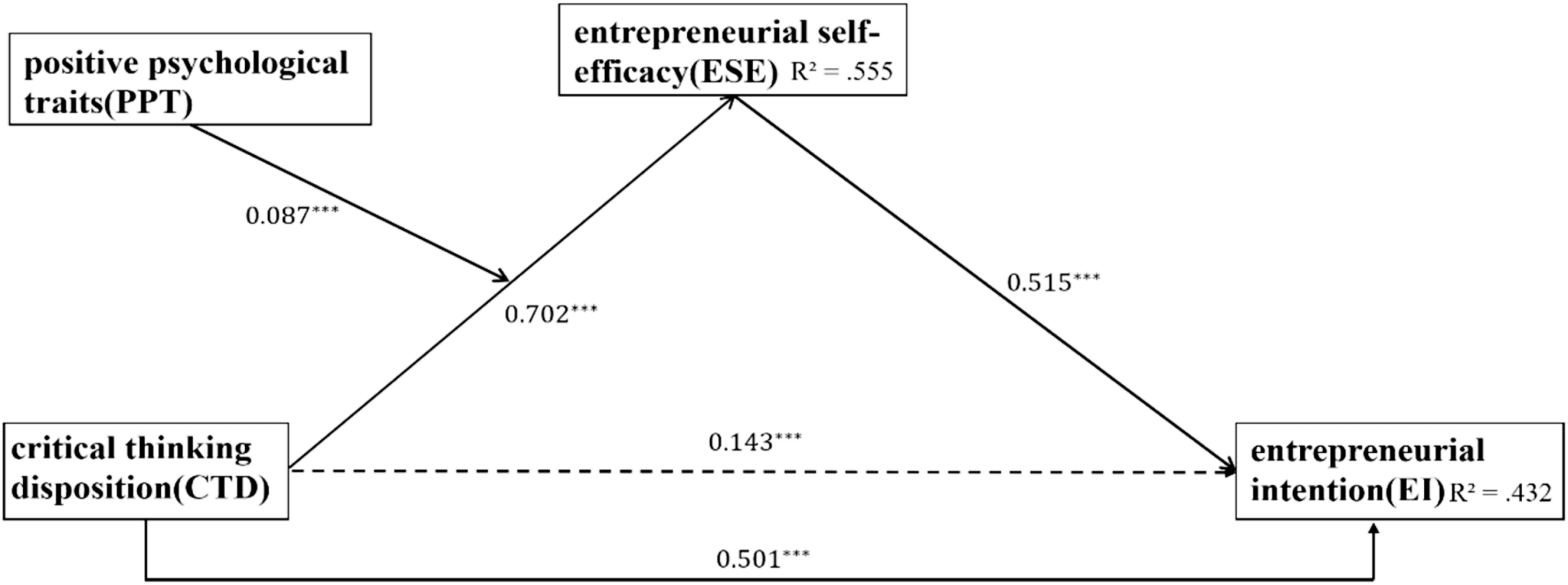
The complete path diagram of the research.

Next, a moderated mediation model was estimated to test whether PPT moderated the mediating pathway. The overall structural model demonstrated an acceptable fit to the data ( $\chi^{2}/\mathrm{df}=23.67$ , CFI = 0.97, TLI = 0.90, RMSEA = 0.079). The model accounted for 43.2% of the variance in the dependent variable EI ( $R^{2}=0.432,\mathrm{SE}=0.031$ ) and 55.5% of the variance in the mediator ESE ( $R^{2}=0.555,\mathrm{SE}=0.031$ ). The complete path diagram with standardized coefficients is presented in Figure 1.

Specifically, results revealed that the interaction between CTD and PPT significantly predicted ESE (*β* = 0.087, *SE* = 0.021, *t* = 4.154, *p* < 0.001, 95% CI [0.045, 0.128]), indicating that PPT strengthened the association between CTD and ESE. However, neither the interaction of CTD × PPT nor ESE × PPT significantly predicted EI (both *ps* > 0.05).

**Figure 2:**
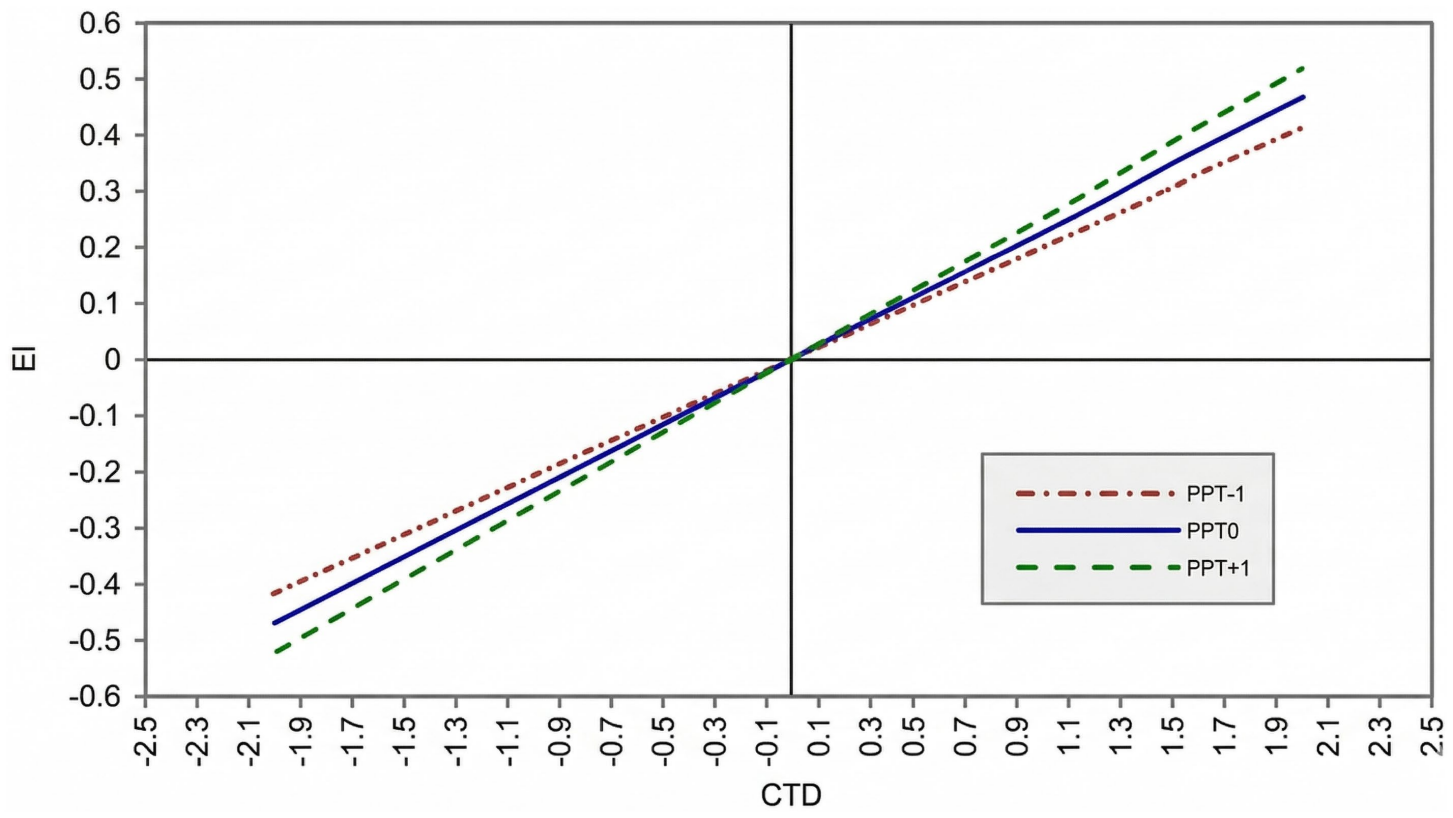
The moderating effect of PPT on the overall association between critical thinking and EI. As indicated in the legend, the dotted line represents the relationship between the independent and dependent variables when PPT are one standard deviation below the mean (−1 SD); the dashed line represents the relationship when PPT are one standard deviation above the mean (+1 SD); and the solid line represents the relationship when PPT are at the mean level (standardized value = 0).

Following the analytic framework proposed by Muller et al. (2005), these results support a significant first-stage moderated mediation effect: PPT moderate the indirect effect of CTD on EI through ESE. Conditional effects analysis further indicated that the indirect effect of CTD on EI increased with higher levels of PPT. Specifically, the indirect effect was 0.209 at −1 SD, 0.230 at the mean, and 0.261 at +1 SD of PPT, with all confidence intervals excluding zero. As shown in Figure 2, the overall association between CTD and EI was strongest at high levels of PPT and weakest at low levels.

## Discussion

### Main findings

The two studies yielded consistent evidence for the role of CTD in shaping EI. Across both samples, students who engaged more readily in analytical reasoning and reflective judgment reported stronger intentions to pursue entrepreneurship. This finding aligns with prior work demonstrating that critical thinking facilitates opportunity recognition and decision-making under uncertainty (Ghafar, 2020; Mary George et al., 2016; Sugito et al., 2020).

The moderating role of PPT, however, showed sample-specific patterns. Among vocational college students (Study 1), positive traits did not significantly alter the relationship between critical thinking and intention. In contrast, the undergraduate sample (Study 2) revealed that students higher in optimism and resilience translated their critical thinking skills into EI more effectively. This suggests that the motivational benefits of psychological capital may depend on educational context or developmental stage (Adomako et al., 2016; Jin, 2017).

Study 2 further clarified the mechanism underlying these effects. Entrepreneurial self-efficacy fully mediated the influence of critical thinking on intention, and this mediation was stronger among students with more positive traits. In other words, critical thinking appears to foster intention primarily by building confidence in one’s ability to execute entrepreneurial tasks, a process amplified by psychological resources.

### Theoretical contributions

This study contributes to entrepreneurship research by clarifying how cognitive dispositions and psychological resources relate to entrepreneurial motivation.

First, the findings extend the TPB by considering critical thinking disposition as an antecedent of perceived behavioral control. Prior applications of TPB in entrepreneurship research have typically treated attitudes, subjective norms, and perceived behavioral control as relatively stable constructs, often measured at a single time point (Krueger et al., 2000; Liñán and Chen, 2009). Such approaches pay less attention to the cognitive processes through which these beliefs develop. The present results suggest that CTD is associated with how individuals evaluate entrepreneurial demands. Students with higher levels of CTD are more likely to engage in systematic evaluation of evidence, recognize underlying assumptions, and consider alternative courses of action. These processes may reduce ambiguity and contribute to a stronger sense of control over entrepreneurial tasks. In this way, CTD can be understood not only as a predictor of intention, but also as a factor related to how individuals interpret and respond to entrepreneurial information. This perspective responds to calls to examine the cognitive antecedents of TPB constructs (Fayolle and Liñán, 2014) and offers a more process-oriented account of belief formation.

Second, the study connects positive psychology with research on entrepreneurial cognition by examining the conditions under which cognitive factors relate to motivation. Existing cognitive approaches to entrepreneurship emphasize information processing (Baron, 2004; Mitchell et al., 2002), but often give less attention to the role of affective and motivational factors. Drawing on the broaden-and-build perspective (Fredrickson, 2001), the results suggest that positive psychological traits may support the translation of critical thinking into self-efficacy. Engaging in critical thinking involves evaluating uncertainty and potential risks, which may also increase cognitive and emotional demands (Paul and Elder, 2006). Traits such as optimism and perseverance may help individuals maintain engagement with these demands and continue reflective analysis. In addition, characteristics such as connectedness and engagement may facilitate learning processes relevant to self-efficacy, including feedback seeking and observation of others. From this perspective, PPT appears to shape how cognitive reflection relates to confidence, rather than simply adding to its effects. This finding suggests that the impact of cognitive skills may vary across individuals depending on their psychological resources.

Third, the results indicate that ESE mediates the relationship between CTD and entrepreneurial intention, and that this indirect relationship varies across levels of PPT. Previous research has identified ESE as a proximal predictor of entrepreneurial intention (Chen et al., 1998; Zhao et al., 2005), but less attention has been given to the factors that contribute to its development. The present findings suggest that critical thinking is associated with higher self-efficacy, possibly because it allows individuals to anticipate entrepreneurial tasks, consider potential challenges, and generate possible responses. These processes may support the development of efficacy beliefs. At the same time, the results indicate that this process depends on individuals’ psychological characteristics. This suggests that the development of self-efficacy is not uniform across individuals and that cognitive skills alone may not be sufficient to support entrepreneurial motivation. Taken together, the findings provide a more integrated account of how cognitive and psychological factors are associated with the formation of entrepreneurial intention.

### Practical implications

These findings also have practical implications for entrepreneurship education where policy initiatives continue to encourage student entrepreneurship and mass innovation (Chen et al., 2021). Programs are likely to be more effective when they develop critical thinking and psychological resources alongside entrepreneurial knowledge. Teaching approaches such as case-based instruction, simulations, and problem-based projects can expose students to ambiguity and trade-offs, encouraging structured analysis and reflective judgment (Samaras et al., 2022). At the same time, educators can embed simple but systematic activities that support optimism, perseverance, and resilience, including goal-setting routines and guided reflection, so that students are better prepared for setbacks and uncertainty (Erikson, 2002; Karaman, 2024; Penggabean et al., 2023).

The results further suggest that curriculum reform should treat ESE as an explicit target rather than an assumed by-product of coursework. Practice-oriented elements that allow students to experience progress and competence, such as entrepreneurship competitions, mentored projects, and sustained contact with entrepreneurs, may provide the mastery experiences that strengthen efficacy beliefs (Shittu, 2017; Zhou et al., 2024). In practice, this implies a coordinated design in which cognitive training in analysis and decision-making is paired with support for psychological resources through advising, workshops, and reflective learning. Such integration may help translate national innovation goals into stronger and more sustained entrepreneurial engagement among students.

### Limitations and future research

This study has several limitations. First, the use of self-reported questionnaire data within a cross-sectional design raises methodological concerns. As all variables were measured at the same time and from the same source, the findings may be affected by CMB, which can inflate the observed relationships among constructs (Podsakoff et al., 2003). In addition, although the measurement models showed acceptable fit, some indices were only moderate. This suggests that the measures may not fully capture the intended constructs (Hu and Bentler, 1999). These issues should be considered when interpreting the results, as they may lead to an overestimation of the strength of the relationships, particularly for the moderating effects. Future research could address these limitations by using data from multiple sources, such as peer or teacher evaluations, and by adopting longitudinal or experimental designs that separate the measurement of predictors and outcomes. Such approaches would allow for stronger causal inferences and more robust measurement (Podsakoff et al., 2012).

Second, the sample was drawn from Chinese vocational and undergraduate students. Although these groups are relevant to current policy discussions, the findings may not generalize to other cultural or educational settings. Future studies should examine whether the observed relationships hold in different countries or among other student populations, as cultural and institutional factors may influence entrepreneurial cognition and intention (Liñán and Chen, 2009).

Third, the study focuses on entrepreneurial intention rather than actual behavior. Prior research has documented a gap between intention and behavior in entrepreneurship (Shirokova et al., 2016). It remains important to examine whether higher levels of intention, associated with cognitive and psychological factors, lead to venture creation or sustained entrepreneurial activity.

Finally, although the EPOCH scale captures several broad positive traits, it is not clear which specific components are most strongly related to the observed effects. Future research may consider examining the roles of individual dimensions, such as optimism or perseverance, in greater detail (Zeng and Kern, 2019). Despite these limitations, the study provides a basis for further research on how cognitive and psychological factors jointly relate to entrepreneurial development.

## Conclusion

In summary, this study suggests that entrepreneurial intention is shaped by both cognitive and psychological factors. Extending beyond traditional predictors, the findings indicate that critical thinking disposition is associated with entrepreneurial intention through its link with entrepreneurial self-efficacy, and that positive psychological traits influence the strength of this relationship. From a theoretical perspective, these results contribute to the Theory of Planned Behavior and Social Cognitive Career Theory by clarifying how cognitive dispositions and psychological resources operate together in the formation of entrepreneurial intention.

In practical terms, the findings imply that entrepreneurship education may benefit from approaches that develop both students’ analytical abilities and their capacity to cope with challenge and uncertainty. At a broader level, promoting such competencies is consistent with the aims of the United Nations Sustainable Development Goals, particularly SDG 4 (Quality Education) and SDG 8 (Decent Work and Economic Growth). Supporting students in developing both cognitive and psychological resources may therefore help address youth employment challenges and contribute to sustainable economic development.

## Data Availability

The raw data supporting the conclusions of this article will be made available by the authors, without undue reservation.
